# Supplementary material for: Chemokines cooperate with TNF to provide protective anti-viral immunity and to enhance inflammation
Source: Nat Commun. 2018 May 3;9:1790. doi: 10.1038/s41467-018-04098-8 (PMC5934441; doi:10.1038/s41467-018-04098-8)
Supplement: Supplementary file 1 — Supplementary Information [file 41467_2018_4098_MOESM1_ESM.pdf]

Chemokines cooperate with TNF to provide protective anti-viral immunity and to enhance inflammation

Alejo et al.

Supplementary Figures

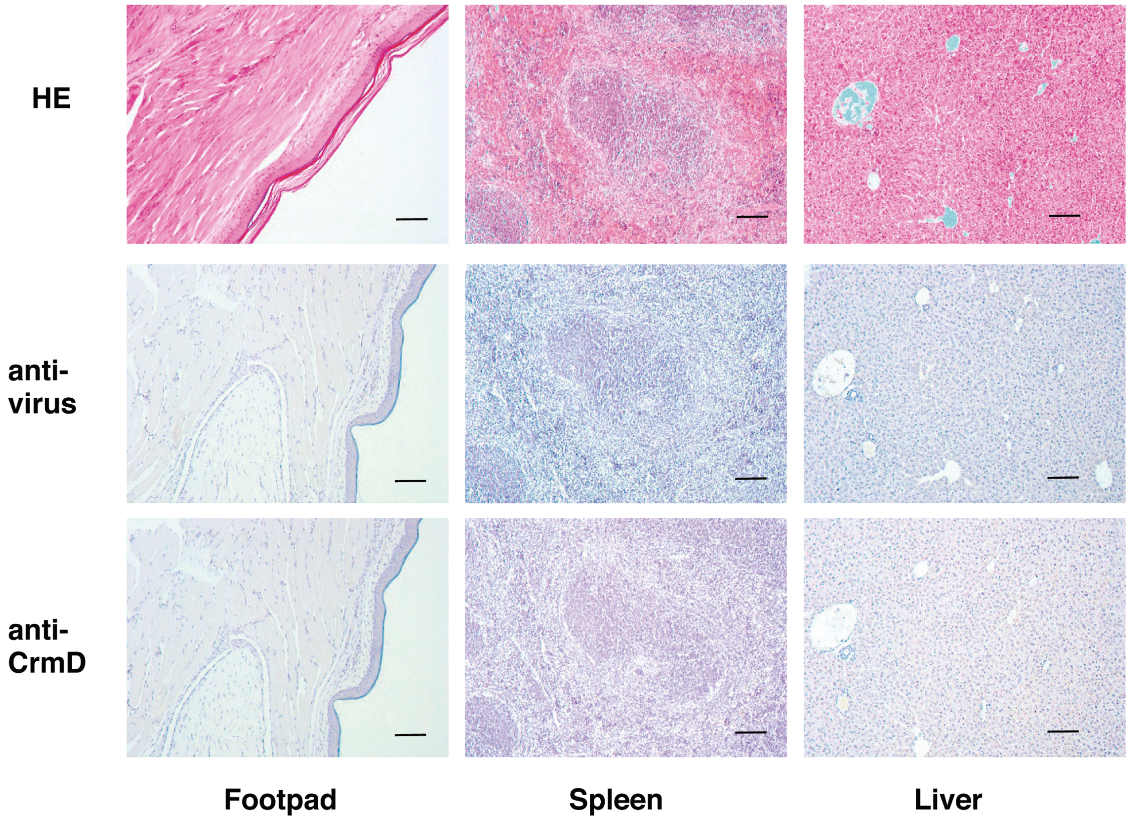

Supplementary Figure 1. H&E staining and immunohistochemistry control of sections from uninfected mice.

Left hind foot, spleen and liver anti-poxvirus (anti-virus) staining or anti-CrmD staining of zinc-fixed sections of representative mock-infected BALB/c mice at 7 dpi. H&E staining of representative sections are also shown. Data are from one experiment representative of two independent experiments. Scale bar, 100 µm.

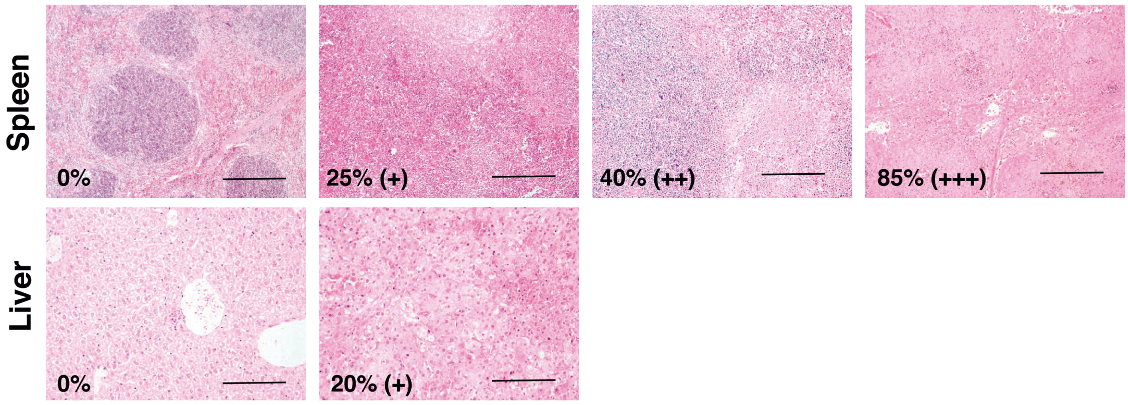

Supplementary Figure 2. Degrees of necrosis in spleen and liver sectoins from ECTV-infected mice.

Representative examples showing the percentage of necrosis estimated. Scale bar, 100 μm.

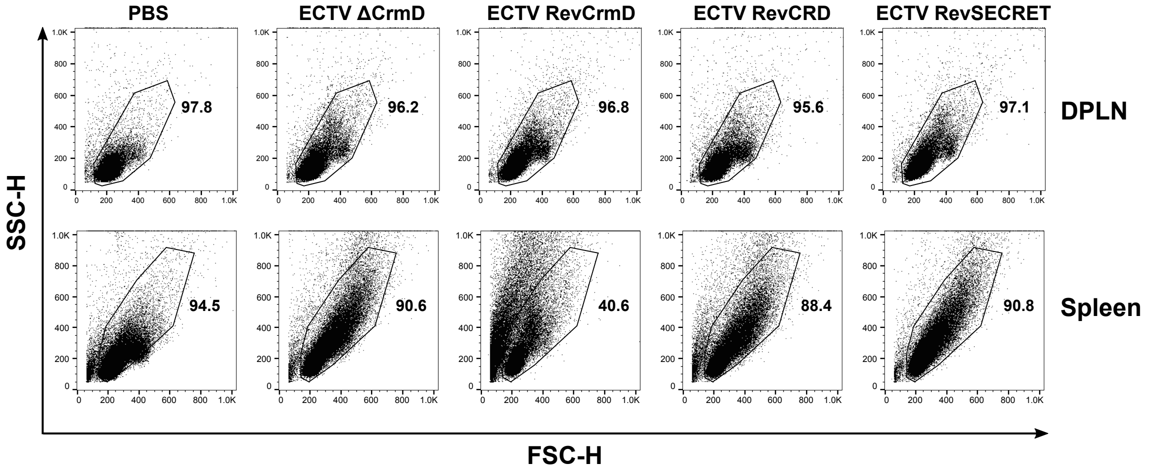

Supplementary Figure 3. Dot-plots showing the FACS gating strategy used for the analyses presented in Figure 8. Cells isolated from DPLN or spleen were gated for a forward and side scatter (FSC and SSC) pattern compatible with healthy leukocytes. Only the events within the depicted gates were included in the analyses. One example for each group (indicated above each graph column) and tissue is shown. Numbers inside each dot-plot indicate the % of gated events over the total population.
